# Data on the role of iba57p in free Fe^2+^ release and O_2_^∙−^ generation in *Saccharomyces cerevisiae*

**DOI:** 10.1016/j.dib.2018.03.023

**Published:** 2018-03-11

**Authors:** Mauricio Gomez-Gallardo, Luis A. Sánchez, Alma L. Díaz-Pérez, Christian Cortés-Rojo, Jesús Campos-García

**Affiliations:** a Lab. de Biotecnología Microbiana, Instituto de Investigaciones Químico Biológicas, Universidad Michoacana de San Nicolás de Hidalgo, Morelia, Mich., Mexico; b Lab. de Bioquímica, Instituto de Investigaciones Químico Biológicas, Universidad Michoacana de San Nicolás de Hidalgo, Morelia, Mich., Mexico

## Abstract

The related study has confirmed that in *Saccharomyces cerevisiae*, iba57 protein participates in maturation of the [2Fe–2S] cluster into the Rieske protein, which plays important roles in the conformation and functionality of mitochondrial supercomplexes III/IV in the electron transport chain (Sánchez et al., 2018) [1]. We determined in *S. cerevisiae* the effects of mutation in the *IBA57* gene on reactive oxygen species (ROS) and iron homeostasis. Flow cytometry and confocal microscopy analyses showed an increased generation of ROS, correlated with free Fe^2+^ release in the *IBA57* mutant yeast. Data obtained support that a dysfunction in the Rieske protein has close relationship between ROS generation and free Fe^2+^ content, and which is possible that free Fe^2+^ release mainly proceeds from [Fe–S] cluster-containing proteins.

**Specifications Table**

**Table:** 

Subject area	*Biology*
More specific subject area	*Cell biology*
Type of data	*Graphs, figures*
How data was acquired	*ROS and Fe*^*2+*^*determination by flow cytometry using a BD Accuri C6 Flow Cytometer (BD Biosciences) and observation by using a confocal microscope (Olympus FV1000).*
Data format	*Analyzed and images*
Experimental factors	*ROS and Fe*^*2+*^*determination in S. cerevisiae cells using fluorescent probes.*
Experimental features	*Real-time quantification of ROS and Fe*^*2+*^*in S. cerevisiae cells suspensions were determined by flow cytometry and cellular structures were co-localizedby confocal microscopy.*
Data source location	*Instituto de Investigaciones Químico Biológicas, Universidad Michoacana de San Nicolás de Hidalgo, Morelia, Michoacán, México.*
Data accessibility	*Data are provided with this article.*

**Value of the data**

• There is an established relation between *IBA57* mutation and the Rieske protein maturation in *S. cerevisiae*, which affects the electron transport chain functionality.
• *IBA57* mutation in *S. cerevisiae* is correlated with ROS generation and loss of iron homeostasis.
• This dataset provides new insights into the mechanism of ROS generation in *S. cerevisiae,* dependent of the ETC functionality.

## 1 Data

**Fig. 1:**
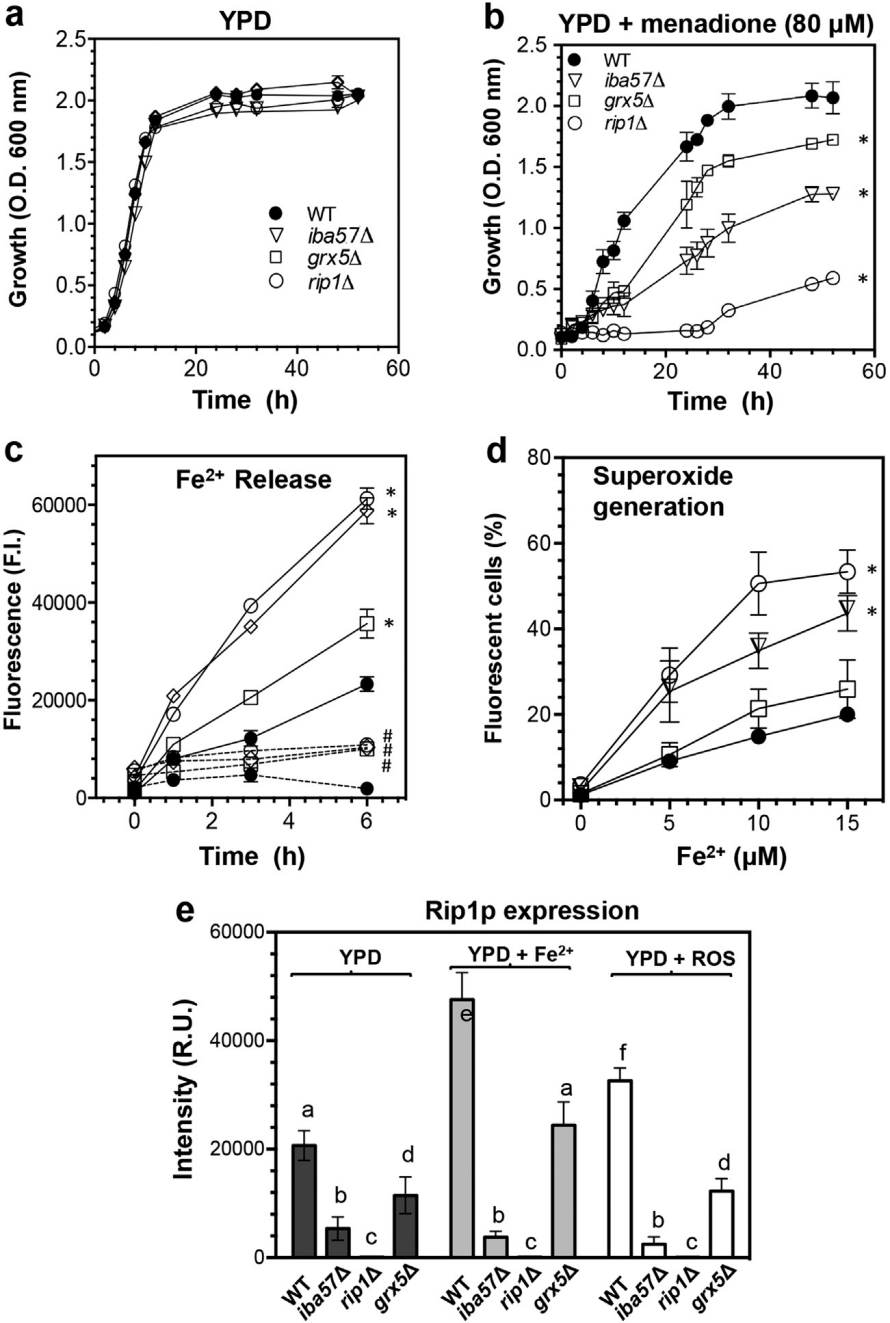
Effect of the *IBA57* deletion over the growth of *Saccharomyces cerevisiae*, iron release, superoxide generation and Rip1 protein expression. a–b) Growth kinetics of *S. cerevisiae* strains grown without and in the presence of menadione 80 μM as ROS-inducer. **c**) Kinetics of Fe^2+^ release. Treatments without menadione (dashed lines) and with 80 μM menadione (continuous lines). d) O_2_^•−^ generation in yeast suspensions treated with different concentrations of Fe^2+^ [FeSO_4_(NH_4_)]. a–d) Values are the mean of three independent experiments. **e**) Densitometry analysis of cellular extracts free-cells immunoblotted for Rip1p expression; yeast extract of cultures grown on: YPD (glucose), YPD with Fe^2+^ [FeSO_4_(NH_4_)] 20 μM, and YPD with menadione 80 μM. Means and SE are indicated as bars (*n* = 3). ANOVA was used to compare treatments. Significant differences (*p* < 0.05) are indicated as symbols (*, #) or with different lowercase letters.

Treatments with 80 µM menadione in the *Saccharomyces cerevisiae iba57Δ* mutant caused significant impairment in its growth rate (Fig. 1a–b). The levels of free Fe^2+^ even without oxidant were significantly incremented in a time-dependent fashion in cell suspensions of the *iba57Δ* mutant yeast (Fig. 1c). The *iba57Δ* mutant displayed a significant increment of superoxide radical (O_2_^•−^) generation with a dose-dependent of Fe^2+^, determined by flow cytometry (Fig. 1d).

The western blot assays showed that the Rieske protein (Rip1p) was absent in the *rip1Δ* mutant, and decreased expression level was found in the *iba57Δ* mutant (Fig. 1e). When extracts from cultures grown on YPD plus high Fe^2+^ concentration (20 µM) or menadione as ROS-inducer were used, the Rip1p expression increased significantly in the WT, but not in the *iba57Δ* mutant.

**Fig. 2:**
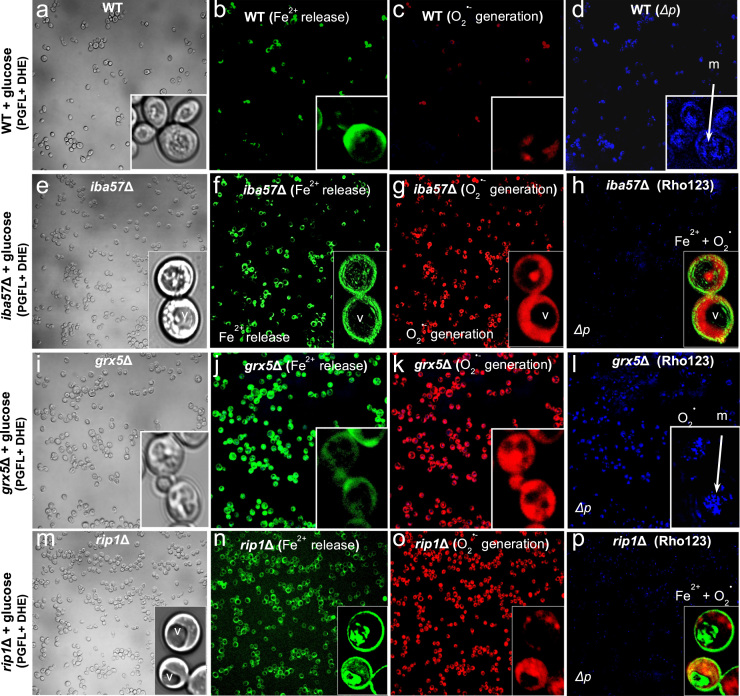
Microscopy images of *Saccharomyces cerevisiae* cells for co-localization of free Fe^2+^ and superoxide in intracellular compartments. YPD-grown yeast cultures were loaded with the fluorescent probes PGFL and DHE for determination of free Fe^2+^ and O_2_^•−^, respectively; incubated for 30 min at 30 °C and co-loaded with Rhodamine 123 for membrane potential (*Δp*) detection as a mitochondrial co-localization marker, and observed using a confocal microscope. a–d) Wild type (WT) yeast; e-h) *iba57*Δ mutant; i-l) *grx5*Δ mutant; and m-p) *rip1*Δ mutant. Cells are shown in which mitochondria and vacuoles are indicated by (m) and (v), respectively. Free Fe^2+^ accumulation is shown as green cells and green granules within the cells, O_2_^•−^ generation areas are shown as red granules within the cells, and mitochondrial structures (*Δp*) are shown as cyan granules within the cells, using the Rho123 probe. Images of the cells were taken at 10× to 60× magnifications using a confocal microscope (Olympus FV1000).

Microscopy analysis shows an increment in ROS generation, associated with release of free Fe^2+^ in the *iba57Δ* mutant (Fig. 2). Interestingly, the high-intensity fluorescence observed in the *iba57Δ* mutant, which exhibited a full dissipation of mitochondrial membrane potential was associated with loss of iron homeostasis in the yeast cells.

## 2 Materials and methods

### 2.1 Yeast strains and growth conditions

Mutant strains *iba57*Δ, *rip1*Δ, and *grx5*Δ correspond to the haploid *S. cerevisiae* BY4741 (Mat a, *his3*Δ, *leu2*Δ0, *met1*5Δ0, *ura3*Δ0) and its *KanMX4* interruption gene (Open Biosystems). Growth tests were carried out as described [1].

### 2.2 Real-time quantification of ROS and Fe^2+^ content in *S. cerevisiae* cultures

Intracellular ROS and Fe^2+^ in cell suspensions were determined using cell-permeant fluorescent probes quantified by flow cytometry [1], [2], [3]. For superoxide (O_2_^•−^) determination, yeast were incubated with 5 µg/mL dihydroethidium (DHE, Molecular Probes, Invitrogen); while as for free Fe^2+^ was used the indicator for heavy metals Phen green FL 5 µg/mL (PGFL; Molecular Probes, Invitrogen) in presence of 1 mM of the chelator 1,10-Phenanthroline (Sigma). DHE- and PGFL-fluorescence was quantified by flow cytometry monitoring the emission fluorescence at 587/40 nm and 533/30 nm, respectively; using a BD AccuriC6 Flow Cytometer (BD Biosciences).

### 2.3 Determination of Rip1p expression by Western blot in *S. cerevisiae*

Mitochondrial protein extracts 50 μg were separated by electrophoresis on SDS-PAGE gels, membranes for Western blot procedure were treated as described [1], [2], [3]. Bands intensity in films were quantified using the Image J software and data graphed as Rip1p expression intensity.

### 2.4 Confocal microscopy of yeast suspensions

*S. cerevisiae* YPD-grown cultures were loaded with the fluorescent probes DHE or PGFL and Rhodamine 123 as detailed [1], [2], [3], treated with menadione (80 µM) and mitochondrial co-localization was analyzed using a confocal microscope (Olympus FV1000). The emission signal of fluorescence was monitored at 560–580 nm for DHE, 405–505 nm for PGFL, and 533–563 nm for Rhodamine 123.
