# Supplementary material for: Data on the role of iba57p in free Fe2+ release and O2∙− generation in Saccharomyces cerevisiae
Source: Data Brief. 2018 Mar 11;18:198–202. doi: 10.1016/j.dib.2018.03.023 (PMC5996255; doi:10.1016/j.dib.2018.03.023)
Supplement: Supplementary file 1 — Transparency document [file mmc1.doc]

Morelia, Mich., México. March 3^th^, 2018.

**Editorial office**

**Data in Brief:**

We declare that this manuscript contains original work and that has not been published elsewhere, it contains additional data that complement to the published paper Mitochondrion. (17)30136-8. doi: 10.1016/j.mito.2018.01.003; by same authors.

All authors are in agreement with the items included in the work presented, and all accept its resubmission to Data in Brief journal.

**The authors: Mauricio Gomez-Gallardo, Luis A. Sánchez, Alma L. Díaz-Pérez, Christian Cortes-Rojo, and Jesús Campos-García, declare that no exist conflict of interests.**

Thank you in advance for your attention and considerations.

Sincerely,


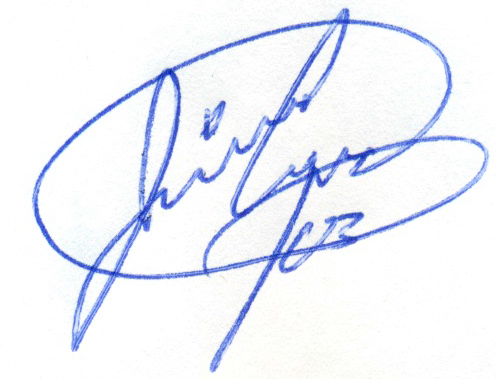


Dr. Jesus Campos Garcia

Corresponding author

Instituto de Investigaciones Químico-Biológicas

Universidad Michoacana de San Nicolás de Hidalgo

Edif. B-3, Ciudad Universitaria, CP 58030, Morelia, Michoacán, México.

Phone/Fax: (52) 443 3265788.

E-mail: jcgarcia@umich.mx
